# Resistant starch consumption promotes lipid oxidation

**DOI:** 10.1186/1743-7075-1-8

**Published:** 2004-10-06

**Authors:** Janine A Higgins, Dana R Higbee, William T Donahoo, Ian L Brown, Melanie L Bell, Daniel H Bessesen

**Affiliations:** 1 University of Colorado Health Sciences Center, Center for Human Nutrition, Denver, Colorado 80262. USA; 2 University of Vermont, Department of Medicine, Burlington, Vermont 05405. USA; 3 University of Wollongong, Wollongong, NSW, 2522. Australia; 4 Preventive & Social Medicine, University of Otago, Dunedin, New Zealand

**Keywords:** resistant starch, fat oxidation, glucose, insulin, amylose

## Abstract

**Background:**

Although the effects of resistant starch (RS) on postprandial glycemia and insulinemia have been extensively studied, little is known about the impact of RS on fat metabolism. This study examines the relationship between the RS content of a meal and postprandial/post-absorbative fat oxidation.

**Results:**

12 subjects consumed meals containing 0%, 2.7%, 5.4%, and 10.7% RS (as a percentage of total carbohydrate). Blood samples were taken and analyzed for glucose, insulin, triacylglycerol (TAG) and free fatty acid (FFA) concentrations. Respiratory quotient was measured hourly. The 0%, 5.4%, and 10.7% meals contained 50 μCi [1-^14^C]-triolein with breath samples collected hourly following the meal, and gluteal fat biopsies obtained at 0 and 24 h. RS, regardless of dose, had no effect on fasting or postprandial insulin, glucose, FFA or TAG concentration, nor on meal fat storage. However, data from indirect calorimetry and oxidation of [1-^14^C]-triolein to ^14^CO_2 _showed that addition of 5.4% RS to the diet significantly increased fat oxidation. In fact, postprandial oxidation of [1-^14^C]-triolein was 23% greater with the 5.4% RS meal than the 0% meal (p = 0.0062).

**Conclusions:**

These data indicate that replacement of 5.4% of total dietary carbohydrate with RS significantly increased post-prandial lipid oxidation and therefore could decrease fat accumulation in the long-term.

## Background

Resistant starch (RS) is any starch that is not digested in the small intestine but passes to the large bowel for fermentation [1]. Retrograded amylose (a linear polymer of glucose residues linked by α(1→4) bonds; RS1), such as cooked and cooled starchy foods like pasta salad, and native starch granules (RS2), such as those found in high-amylose maize starch and bananas, are the major components of dietary RS. Calories from RS that are undigested in the small intestine can be salvaged by fermentation to short-chain fatty acids (SCFA; acetate, butyrate, proprionate) by the microflora of the large bowel. Fermentation of RS in the large bowel gives rise to increased production of SCFA which is reflected in higher epithelial and portal concentrations. SCFA concentration in the periphery, however, is very low and therefore difficult to measure accurately so any increase in production of SCFA in response to RS consumption may not be detectable in the peripheral circulation.

Acute human studies describe variable postprandial glycemic and/or insulinemic responses to RS ingestion. In general, it is accepted that RS consumption lowers postprandial glucose concentrations marginally and postprandial insulin concentrations markedly. Many groups report a decrease in postprandial glycemic or insulinemic responses to RS ingestion relative to digestible starch (DS) consumption [2-7], whereas some report no change [8-11]. It is important to note that the fat content of the diet has a significant impact on the glycemic response to a meal and some meal tests contained no fat or the fat content of the meal varied among the different RS diets making results from these studies difficult to interpret [2-4]. Also, there are many sources of RS, such as beans, high amylose corn starch, and potatoes, which possess different physicochemical properties. So, the source of RS can influence the glycemic/insulinemic response to RS ingestion.

Many studies have examined the relationship between RS ingestion and postprandial metabolite and hormone concentrations. Fewer studies have documented the effect of RS on lipid metabolism. In humans, five weeks of RS feeding lowered fasting cholesterol and triglyceride concentrations and postprandial plasma insulin concentrations relative to digestible starch (DS) feeding [12,13]. It has also been reported that chronic RS feeding in rats causes a decrease in adipocyte cell size relative to DS feeding [14,15]. In addition, expression of fatty acid synthase was lower in rats fed a RS-based diet than in those fed a DS-based diet [16]. Taken together, these studies provide evidence that RS intake has an effect upon the activity of key lipogenic enzymes and adipocyte morphology. Thus, it seems that the effects of this carbohydrate subtype on lipid metabolism should be carefully examined in human studies.

It is possible that strong physical association between RS and dietary lipid may slow the absorption, and thereby increase the oxidation, of dietary lipid. Currently, there is no evidence pertaining to the dose-response relationship for RS ingestion (as part of a mixed meal) and postprandial glycemia, insulinemia, fat oxidation, or meal fat storage. It is important that these parameters be defined before designing and conducting long-term, prospective RS feeding studies.

## Results

**Figure 1:**
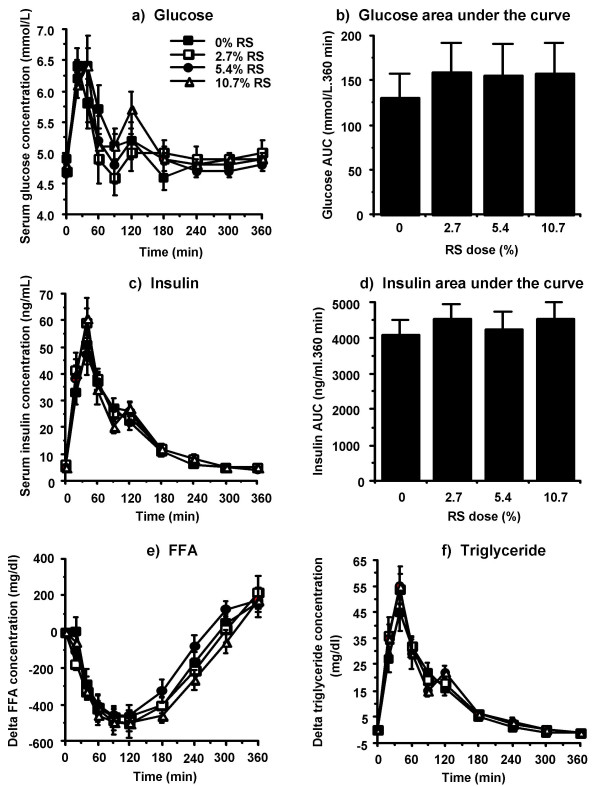
**Circulating glucose (a, b), insulin (c, d), free fatty acid (e), and triglyceride (f) concentrations in response to the RS content of a breakfast meal. **Serum glucose and insulin measurements were conducted on 12 healthy adults. Data is presented as mean ± SEM.

No difference in fasting or postprandial insulin, glucose, FFA, or triglyceride concentration was observed between any of the RS doses examined (Figure 1).

**Figure 2:**
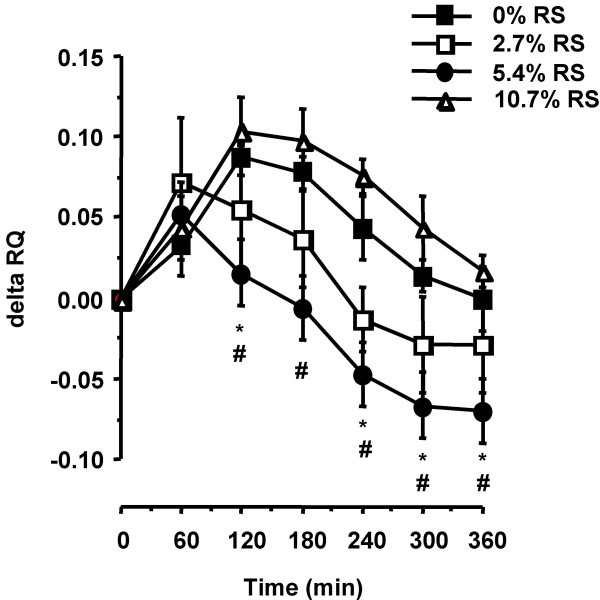
**Respiratory quotient (RQ; change from baseline) in response to RS content of a breakfast meal. **Respiratory gas exchange measurements were conducted on 12 healthy adults using the ventilated hood method. Data is presented as mean ± SEM. * p < 0.05 for a difference from the 0% meal at the same time point. # p < 0.03 for a difference with the 10.7% meal at the same time point.

**Figure 3:**
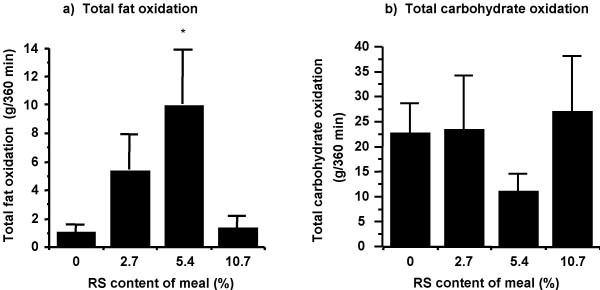
**Total fat (a) and carbohydrate (b) oxidation in response to RS content of a breakfast meal. **Macronutrient oxidation, assessed via indirect calorimetry and calculated from non-protein RQ, was measured in 12 healthy adults. Data is presented as mean ± SEM. * p ≤ 0.003 for a difference from the 0% and 10.7% RS meals.

Overall, the dose of RS in the meal had a significant influence on ΔRQ (respiratory quotient) values (F-test, 0.04; Figure 2). This overall effect was due to a significantly lower ΔRQ at the 5.4% RS dose than the 0% (p = 0.02) or 10.7% (p = 0.009) RS doses, indicating an increase in fat oxidation in response to the 5.4% RS meal relative to the 0% and 10.7% RS doses (Figure 2). ΔRQ was significantly lower for the 5.4% RS meal than 0% RS meal at 120, 240, 300 and 360 minutes (p = 0.05, 0.03, 0.02 and 0.04, respectively) whereas significant differences occurred at 120, 180, 240, 300 and 360 minutes (p = 0.01, 0.01, 0.005, 0.02, and 0.03, respectively) for the 5.4% RS versus 10.7% RS meals. These data are reflected in total macronutrient oxidation rates (Figure 3), which show a significant increase in the amount of fat oxidized at the 5.4% RS dose relative to the 0% RS meal, with a concomitant decrease in total carbohydrate oxidation.

**Figure 4:**
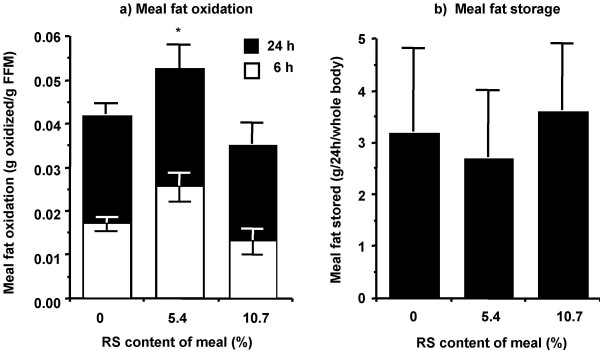
**Meal fat oxidation (a) and storage (b) in response to RS content of a breakfast meal. **Meal fat oxidation, assessed via measurement of ^14^CO_2 _in expired air, and meal fat storage in gluteal adipose tissue was measured in 12 healthy adults. Data is presented as mean ± SEM. * p ≤ 0.0006 for a difference from the 0% and 10.7% RS meals at the same time point. FFM, fat free mass.

Similarly, the oxidation of [^14^C]-triolein to ^14^CO_2 _was different between RS doses (F-test, 0.0005). Meal fat oxidation at the 5.4% RS dose was significantly higher than both the 0% (p = 0.0062) and 10.7% doses (p < 0.0001). Separate tests at 6 h or 24 h following the test meal gave comparable results (Figure 4a). Taken together, these independent measurements of fat oxidation (indirect calorimetry, oxidation of [^14^C]-triolein to ^14^CO_2_) suggest that the inclusion of 5.4% RS in the meal elevated postprandial fat oxidation. Unexpectedly, this effect was lost if the dose was increased to 10.7% RS.

There was a trend for fat storage from the test meal, as assessed by incorporation of ^14^C into gluteal adipose tissue, to be lower for the 5.4% RS meal than all other meals, although this effect did not reach statistical significance (Figure 4b).

## Discussion

This study demonstrated that the addition of RS to a mixed meal, balanced for total fat and fiber content, had no effect on postprandial glucose, insulin, FFA, or triglyceride excursions. However, meals containing a moderate amount of RS caused an increase in fat oxidation as measured by both indirect calorimetry and the production of ^14^CO_2 _from a ^14^C-triglyceride tracer. Unexpectedly, the dose-response relationship between RS content of the diet and fat oxidation was not linear. Although this result is difficult to explain in the current context, it emphasizes the need for careful selection of RS dose in prospective feeding studies.

There was no difference in postprandial glucose (Figure 1a), FFA (Figure 1e), triglyceride (Figure 1f), or insulin (Figure 1c) concentrations at any RS dose examined. This concurs with data from other acute human studies using complete, mixed meals which showed no difference in postprandial glycemia/insulinemia in response to RS content of the diet [8-11]. Although this seems contrary to the general perception that RS ingestion reduces postprandial insulinemia and glycemia, many of the studies indicating this did not balance test diets for total fat and/or fiber content [17]. However, in the current study all diets were carefully matched for total fat and fiber content. This an important distinction between this and other studies as fiber has extensively been shown to reduce postprandial glycemia/insulinemia and increasing the RS content of the diet intrinsically increases the total fiber content. Also, dietary fat can have potent effects on the accessibility of dietary carbohydrate to digestive enzymes and on the rate of gastric emptying/gut motility. Thus, the glucose- and insulin-lowering effects of RS that have been observed in other studies may be due to changes in fiber and/or fat between test meals which have been extensively shown to lower postprandial glycemic and insulinemic responses. So, the balanced conditions used in the meal tests for the study described herein, which included baked products and processed foods as part of a complete, mixed meal, balanced for total fat and fiber content, could account for the lack of difference in insulinemia and glycemia in response to increased RS content in the diet.

Both indirect calorimetry and ^14^C-tracer data indicate that there was an increase in fat oxidation between the 0% and 5.4% RS doses (Figures 2, 3, and 4a). This increase in total and meal fat oxidation in response to the 5.4% RS meal is not driven by disparate responses amongst subjects as 11 of the 12 subjects studied showed the greatest fat oxidation in response to the 5.4% RS meal, relative to the 0% and 10.7% RS meals (see Figure S1, Additional File 1, for individual responses). Tracer data showed that the addition of 5.4% of RS to a meal increased meal fat oxidation by more than 20% over the 6 h and 24 h post-meal ingestion period (Figure 4a). The increase in fat oxidation at 6 h accounted for approximately one-half of the total increase over 24 h, indicating that the increase in meal fat oxidation in response to a single meal containing 5.4% RS is a prolonged, sustained effect. In addition, comparison of total and meal fat oxidation (Figures 3a and 4a) indicates that endogenous fat stores were the predominant source of fat utilized for energy, contributing approximately 80% of the total fat oxidized, with a much lower contribution from ingested meal fat. Figure 3 shows that this increase in fat oxidation at the 5.4% RS dose is accompanied by a relative reduction in carbohydrate oxidation (does not reach statistical significance).

The increase in fat oxidation at the 5.4% RS dose relative to the 0% dose was not driven by any disparity in circulating glucose, insulin or FFA concentration (Figure 1; see Figures S2, S3, S4, Additional Files 2, 3, 4, respectively, for individual subject responses) nor by a difference in available carbohydrate between the 0% and 5.4% RS meals. If decreased carbohydrate availability was responsible for the observed increase in fat oxidation, the 10.7% RS meal, which has the least available carbohydrate, would show the greatest increase in fat oxidation. However, there was no difference in fat oxidation between the 0% and 10.7% RS meals. Thus, carbohydrate availability cannot be a contributing factor to the increase in fat oxidation observed at the 5.4% dose of RS. It is possible that this increase may be due to an increase in circulating SCFAs from the fermentation of RS reaching the large bowel. The observed increase in fat oxidation is not due to oxidation of these SCFAs *per se *as it was measured directly from conversion of ^14^C-labeled meal fat to ^14^CO_2 _(Figure 3a). Such a measurement would not detect any increase in SCFA oxidation. Rather, it may be that the metabolic effects of increased SCFA production cause an increase in fat oxidation.

RS consumption has been shown to alter the acetate:butyrate:propionate ratio compared to fermentation of non-starch polysaccharides [29]. In particular, the amount of butyrate is substantially elevated in response to RS fermentation [30,31]. In humans fed a low or high RS diet for three days, the concentration of excreted SCFA rose from 20 mmol/d to 33 mmol/d, respectively [19]. This increase in total SCFA concentration was caused by a doubling of the acetate and butyrate content changing the acetate:butyrate:propionate ratio from 12:3:3 to 21:6:4 in response to the low and high RS diets, respectively.

In vitro data from isolated animal tissues provide convincing evidence for the role of SCFAs in carbohydrate and lipid metabolism [26,32-34]. Acetate and/or butyrate have been shown to decrease glycogenolysis and glycolysis in isolated rat and sheep hepatocytes [35-37]. So, it is plausible that the fermentation of RS from the 5.4% RS diet increases the net production of SCFAs which inhibit glycolysis in the liver. In this scenario, the liver, deprived of carbohydrate-derived acetyl CoA would be more reliant on fat-derived acetyl CoA as a fuel source, thereby contributing to an overall increase in fat oxidation [17]. This possibility needs to be investigated in future studies.

No difference in fat oxidation was evident between the maximal 10.7% dose of RS and the 0% dose. This is an unexpected result that is difficult to explain. The loss of any effect on fat oxidation when the RS dose in the meal was increased to 10.7% may occur because this dose is at the threshold of the starch's properties as RS. That is, at the 10.7% dose of RS, the starch may not be completely fermented in the large bowel thereby causing a loss of energy from the diet via the feces. If this is the case, the strong physical association between RS and dietary lipid may cause excretion of lipid and therefore, less dietary fat to be available for oxidation at the 10.7% dose. Indeed, it has previously been shown that intake of high-amylose maize starch, such as that used in this study, caused an increased number of bowel actions per day [18]. RS has also been shown to decrease colonic transit time and, as more RS enters the large bowel, more starch is also excreted [19,20]. This indicates that, at higher levels of RS consumption, only a portion of the RS can be fermented and the remainder passes through the colon as an insoluble fiber. Furthermore, if indeed RS at the 10.7% dose is being excreted as insoluble fiber, less fermentation and SCFA production would be occurring. As SCFA are hypothesized to be the cause of the observed increase in fat oxidation in response to the 5.4% RS meal, this would have a large impact on the fat oxidation potential of the 10.7% RS diet.

The hypothesis that RS is acting like dietary fiber and being excreted can be tested by measuring the amount of fat excreted in the feces. As this outcome was not predicted, fecal samples were not collected from subjects during this study. It is important to consider that it is difficult to add 10.7% RS to a standard diet without the use of specially designed foods and/or without significantly increasing caloric intake. Therefore, this level would be difficult to attain in a free-living situation and the lower doses used in this study are more reflective of predicted levels if normal, starchy foods in the diet were to be replaced with commercially available RS products.

In addition, not all biological processes display linear dose-response curves. Dose-response curves can vary from sigmoidal to 'U'-shaped curves for processes as diverse as drug absorption/clearance [21], low dose radiation effects on cells [22], DNA repair following double-strand breaks [23], and metabolic parameters. Metabolic processes that are non-linear functions include the level of illuminance and plasma melatonin levels [24], caffeine intake versus plasma caffeine metabolite concentrations [25], allergen exposure (concentration) and histamine response [26], zinc-stimulated histamine release from mast cells [27], and fructose-1,6-diphosphate metabolism in cardiomyocytes [28]. Thus, it is possible that the lack of any effect on fat oxidation at the 10.7% RS dose may indicate that the relationship between RS intake and fat oxidation is indeed a 'U'-shaped curve. However, more RS doses between 5.4% and 12% must be tested to accurately define the shape of this dose response curve.

It must be noted that the calculation of oxidation of [^14^C]-triolein via measurement of ^14^CO_2 _did not take into account the dilution of tracer *in vivo *due to the incorporation of labeled carbons into intermediates of the TCA cycle and endogenous bicarbonate pools. Generally, an acetate correction factor is used to account for this effect. In this study, subjects consumed all four test meals under the same conditions and it was assumed that there was no difference in tracer recovery between tests. Also, these TCA intermediate and bicarbonate pools were not pre-labeled prior to the ingestion of the label in the meal which would cause a total underestimation of total fat oxidation. Therefore, the rate of fat oxidation calculated from ^14^CO_2 _recovery in the breath was probably underestimated in all subjects but remains valid to compare differences between test meals.

There was a trend towards a decrease in gluteal fat storage at the 5.4% RS dose relative to all other doses (Figure 4b). Again, the dose-response curve for this parameter was not linear, lending credence to the idea that the dose-response curve for fat oxidation is actually U-shaped. Although the decrease in fat storage at the 5.4% RS dose did not reach statistical significance, it is intuitive that, given the magnitude of the increase in fat oxidation observed at this dose, there would be a reciprocal decrease in fat storage. However, there was high variability associated with the measure of meal fat storage indicating that more subjects may be needed to decrease the standard deviation and, hence, detect any significant meal affect.

## Conclusion

This study is the first to identify that addition of 5.4% RS to a single meal can cause a significant increase in total and meal fat oxidation in healthy individuals relative to a 0% RS diet over the postprandial/postabsorptive period (24 h). This discovery was verified using two different methods, indirect calorimetry and the oxidation of [^14^C]-triolein to ^14^CO_2_, to measure *in vivo *fat oxidation. This increase in fat oxidation was accompanied by a concomitant decrease in carbohydrate oxidation and fat storage, although these parameters did not reach statistical significance. Further, the magnitude of the increase in fat oxidation indicates that this effect is biologically relevant and could be important for preventing fat accumulation in the long term by effecting total fat balance under chronic feeding conditions. Finally, this study revealed that there may be a maximal effect of RS addition to the diet and that the addition of RS over this threshold confers no metabolic benefit or change from a 0% RS meal.

## Methods

### Subjects

12 healthy adults, 7 male and 5 female, participated in the present study. This study was approved by the Colorado Multiple Institution Review Board, in compliance with the Helsinki Declaration, and full written consent was obtained from all subjects. To participate, subjects were required to be between 28 and 45 years of age, have normal glucose tolerance (as judged via response to an oral glucose tolerance test; fasting glucose concentration < 6 mM, postprandial glucose concentration not higher than 9 mM), moderate level of physical activity (no more than 4 one-hour bouts of planned physical activity per week), and a BMI between 20 and 28. All female subjects were taking oral contraceptive pills or progesterone injections and were tested during the early follicular phase of the menstrual cycle. All subjects underwent dual energy X-ray absorptiometry (DEXA; Lunar Radiation Corp, Madison WI) for analysis of body composition. As a group, subjects were 33 ± 5 years of age, 1.7 ± 0.07 m tall, weighed 75 ± 11 kg, had a BMI of 24.7 ± 2.4, total fat mass of 18.3 ± 5.0 kg (mean ± SD), and a fasting RQ of 0.750 ± 0.023 (mean ± SEM).

### Diet

Subjects received four meals differing only in resistant starch (RS) content in random order, approximately four weeks apart. Test meals contained either 0%, 2.7%, 5.4%, or 10.7% RS as a percentage of total dietary carbohydrate. All added RS was in the form of high-amylose maize starch, or RS2. High-amylose maize starch was chosen as it has the unique property of a very high gelatinisation temperature which allows it to maintain its granular structure during and after the processing conditions used to manufacture the foods being consumed in this study [38].

All meals were isocaloric, accounting for 30% of the subject's daily energy needs as measured by indirect calorimetry prior to study commencement (RMR × daily activity factor of 1.49). The composition of the test diet was 55% carbohydrate, 15% protein, and 30% fat as a percentage of total energy (Table 1). All meals were matched for total dietary fiber content and liquid volume (250 ml).

**Table 1:** Composition of test breakfasts. All values are based on a hypothetical subject who requires 8374 kJ (2000 kcal) per day.

RS content (% total carbohydrate)	0	2.7	5.4	10.7
RS content (g)^1^	0 g	2.5 g	5 g	10 g
Total energy (kJ)	2508	2506	2500	2506
Carbohydrate (g)	93.8	93.3	92.9	93.0
Protein (g)	22.7	22.6	23.0	23.0
Fat (g)	17.0	16.8	16.9	16.9
Total sugars (g)	45.6	45.2	45.7	45.1
Total Fiber (g)	9.4	9.3	9.5	9.5
Liquid volume (mL)	250	250	250	250
Foods consumed (g)				
Canned spaghetti	197	58		
*RS Canned spaghetti		147	218	216
Parmesan cheese	10	8	8	12
Margarine	4	3	2	2
Butter	2	1		1
Milk (2% fat)	250	250	210	
*Up & Go breakfast drink			40	250
Bread	38	44	36	
*Banana muffin				43
Strawberries	203	162	123	
Grapes	80	93		
*Fruit fingers			15	16
Sugar, white			10	

* Denotes foods with added RS.

^1 ^Absolute RS inclusion varied according to the energy needs of the subject so that RS content always remained the same fraction of total dietary carbohydrate, namely 0%, 2.7%, 5.4%, and 10.7% for the 0 g, 2.5 g, 5 g, and 10 g meals, respectively. For example, a subject who had a daily caloric need of 9421 kJ (2250 kcal) would receive meals containing 0 g, 2.7 g, 5.4 g, and 10.8 g RS.

^2 ^Energy and macronutrient values were determined using the USDA Nutrient Database for standard foods and from information supplied by the manufacturer for foods with added RS. Note that energy values calculated from the carbohydrate, fat, and protein content of study foods using the 4-9-4 kcal/g factor method differ from reported energy values due to use of the Atwater system.

Three days prior to each test day, subjects received a standardized lead-in diet, equivalent to daily energy needs as judged by indirect calorimetry and of the same macronutrient composition as the test diet with no added RS, to ensure that they were in energy balance. All food for these three days was provided by the General Clinical Research Center (GCRC) on an outpatient basis. Subjects were instructed to eat all of the food/drink provided and not to consume any other foods. Non-caloric beverages could be consumed during the three day lead-in diet.

### Protocol

Following an overnight fast (12 h), subjects were admitted to the GCRC and an intravenous catheter was placed for the purposes of drawing blood. The test meal began at 0 min (0800 h) with all food/drink fully consumed within 15 min. Blood samples were taken at 0, 30, 60, 90, 120, 180, 240, 300, and 360 min following meal ingestion and analyzed for glucose, insulin, triacylglycerol (TAG) and free fatty acid (FFA) concentrations. Respiratory quotient (RQ) was measured at hourly intervals after ingestion of the meal via gas collection under a ventilated plexiglass hood for 15 min (Sensormedics 2900 metabolic cart). All urine produced between 0 and 360 min was collected and analyzed for nitrogen content by the GCRC Core Laboratory to facilitate calculation of non-protein RQ.

In three of the test meals (0%, 5.4%, and 10.7% RS meals), the bread product in the test meal was spiked with 50 μCi [1-^14^C]-triolein (glycerol tri [1-^14^C]oleate; Amersham Pharmacia Biotech, Amersham, UK) suspended in olive oil and the tests were conducted as 24 h inpatient stays at the GCRC. The fat tracer was fed as a triglyceride (glycerol tri [1-^14^C]oleate) rather than a FFA (eg. [1-^14^C]oleate) in order to reflect any change in the absorption of triglyceride FFA which might be due to a strong physical association with RS thereby slowing absorption. At hourly intervals following the meal, then at 8, 10, 12, 14 and 24 hours, breath samples were collected via exhalation through a tube with a one-way valve into scintillation vials containing 2 mmol benzethonium hydroxide (to trap 2 mmol CO_2_), 1 ml methanol, and 1 mg phenolpthalene as a pH indicator. Gluteal fat biopsies were collected by aspiration through a 14 g stainless steel needle at baseline and 24 h after ingestion of the test meal. All breath and fat samples were assayed for the presence of ^14^C (as described below). For these 24 h tests, subjects received 30% of daily energy needs at each of breakfast, lunch, and dinner, with the remaining 10% of calories received in an evening snack. The timing of meals/snacks was kept constant over all tests. All food was provided by the GCRC on an inpatient basis and the macronutrient content of each meal was the same as that of the test meal. Only the test breakfast contained RS during these 24 h tests, all other meals were composed of standard, commercially available products.

### Analyses

All glucose, FFA, and TAG assays were conducted by the GCRC Core Laboratory using an automated Cobas Mira Plus (Roche Diagnostics, Basel, Switzerland). Serum insulin measurements were also performed by the GCRC Core Laboratory using a human insulin RIA kit (Linco, St. Louis, USA).

Fat samples, frozen in liquid nitrogen and stored at -80°C until processing, were incubated in 450 μl Solvable (Packard Bioscience, Groningen, Netherlands) at 50°C for 12 h before the addition of 100 μl 30% (v/v) hydrogen peroxide (for sample bleaching). Fat samples were counted in Aquasol (Packard Bioscience, Groningen, Netherlands) whereas breath samples were counted in Scintisafe 30% (Fisher Chemical, New Jersey) using a Beckman LS6500 scintillation counter (Beckman Instuments, Fullerton, CA). After scintillant was added, all samples were kept in the dark at room temperature for 48 h before being counted to reduce chemiluminescence.

### Calculations

#### Calculation of total fat and carbohydrate oxidation

Formulae used to calculate non protein RQ and subsequent estimations of carbohydrate and fat oxidation were based on the derivations described by Jéquier *et al*. ([39]).

#### Calculation of ΔRQ

ΔRQ = RQ_t _- RQ_baseline_

where t is sample time (min).

#### Calculation of meal fat storage from biopsy data

μg fat stored/g fat tissue = (dpm_24h_/g tissue weight) - (dpm_baseline_/g tissue weight) × 1/specific activity

μg fat stored/whole body = μg fat stored/g fat tissue × total fat mass (from DEXA)

#### Calculation of ^14^C-triolein oxidation

counts from sample (dpm/mol CO_2_)/vCO_2 _(min.ml) = (dpm_t _- dpm_background_) × 1/vCO_2 _=

dpm.mol CO_2_/ min.ml

dpm/min = dpm.mol CO_2_/ min.ml × 0.446 (as 1 ml CO_2 _= 0.446 mol)

g fat oxidized = AUC(dpm/min) × 1/specific activity

where vCO_2 _is the rate of CO_2 _production as assessed during indirect calorimetry. t is sample time (min). AUC is the incremental area under the curve.

### Analysis

All statistical analyses were performed using the statistical analysis software SAS, version 8.1 (SAS OnlineDoc, 2000) with a significance level of p = 0.05 and p = 0.01 for interaction terms. All results are presented as mean ± SEM, except for subject characteristics which are described as mean ± SD. To investigate each of the outcomes (glucose, insulin, FFA, TAG, RQ, meal fat oxidation, and meal fat storage) we used a mixed model with fixed effect terms for RS DOSE, TIME and the interaction of the two, RS DOSE*TIME. Subjects were included as random effects. The interaction term was not significant for any of the outcomes tested so an additive model was used to test the overall effect of RS DOSE and the differences between doses. To test the effects of RS DOSE at different TIMES, a model that included RS DOSE, TIME and RS DOSE*TIME was used. The repeated measures nature of the study design was taken into account by using the covariance structures available in SAS PROC MIXED. For example, measurements within a subject are assumed to be more highly correlated than between subjects, and within a particular treatment, within a subject, the measurements are assumed to be more correlated. Measurements closer in time to one another were modeled with an autoregressive, or AR(1) covariance structure.

## Abbreviation List

RS, resistant starch; DS, digestible starch; TAG, triacylglycerol; FFA, free fatty acid; FFM, fat free mass; SCFA, short-chain fatty acids; GCRC, General Clinical Research Center; RQ, respiratory quotient

## Competing interests

Janine Higgins and Ian Brown are listed as inventors on RS patents filed by Penford Australia Limited. Both Drs. Higgins and Brown are listed as inventors on these patents as they have intellectual property ownership of some of data used in these but receive no financial benefit.

## Authors' Contributions

JH conceived of the study design and was responsible for overall study coordination, conducting patient visits, data analysis, and manuscript preparation. DH was responsible for patient scheduling, day-to-day study coordination, conducting patient visits, and data entry. WD contributed to the study design and manuscript preparation, and conducted patient physical examinations and fat biopsies. IB contributed to the study design, selection of RS foods, and assisted with manuscript preparation. MB conducted all statistical analysis. DB contributed to the study design and manuscript preparation, and conducted patient visits, patient physical examinations and fat biopsies. All authors read and approved the final manuscript.

## Supplementary Material

Additional File 1 **Individual meal (a) and total fat oxidation (b) in response to the RS content of a test breakfast. **Meal fat oxidation, assessed via measurement of ^14^CO_2 _in expired air, and total fat oxidation, assessed via indirect calorimetry and calculated from non-protein RQ, and was measured in 12 healthy adults. Click here for file

Additional File 2 **Individual area under the glucose curve vs. meal (a) and total fat oxidation (b) in response to a test breakfast. **Meal fat oxidation, assessed via measurement of ^14^CO_2 _in expired air, and total fat oxidation, assessed via indirect calorimetry and calculated from non-protein RQ, and was measured in 12 healthy adults. Data from all three test meals (0%, 5.4%, and 10.7% RS) is shown. The relationship between area under the glucose curve and fat oxidation remains the same (i.e. no relationship) when represented as individual doses or, as in this plot, for all doses (see Figure S3). Click here for file

Additional File 3 **Individual area under the glucose curve vs. meal fat oxidation in response to a 0% (a) or 5.4% (b) RS test breakfast. **Meal fat oxidation, assessed via measurement of ^14^CO_2 _in expired air, and total fat oxidation, assessed via indirect calorimetry and calculated from non-protein RQ, and was measured in healthy adults. Data from individual test meals is shown. Click here for file

Additional File 4 **Individual area under the insulin curve vs. meal (a) and total fat oxidation (b) in response to a test breakfast. **Meal fat oxidation, assessed via measurement of ^14^CO_2 _in expired air, and total fat oxidation, assessed via indirect calorimetry and calculated from non-protein RQ, and was measured in 12 healthy adults. Data from all three test meals (0%, 5.4%, and 10.7% RS) is shown. (Document type: Powerpoint, PPT) Click here for file
