# Supplementary material for: Resistant starch consumption promotes lipid oxidation
Source: Nutr Metab (Lond). 2004 Oct 6;1:8. doi: 10.1186/1743-7075-1-8 (PMC526391; doi:10.1186/1743-7075-1-8)
Supplement: Additional File 1 — Individual meal (a) and total fat oxidation (b) in response to the RS content of a test breakfast. Meal fat oxidation, assessed via measurement of 14CO2 in expired air, and total fat oxidation, assessed via indirect calorimetry and calculated from non-protein RQ, and was measured in 12 healthy adults. [file 1743-7075-1-8-S1.ppt]

## Slide 1
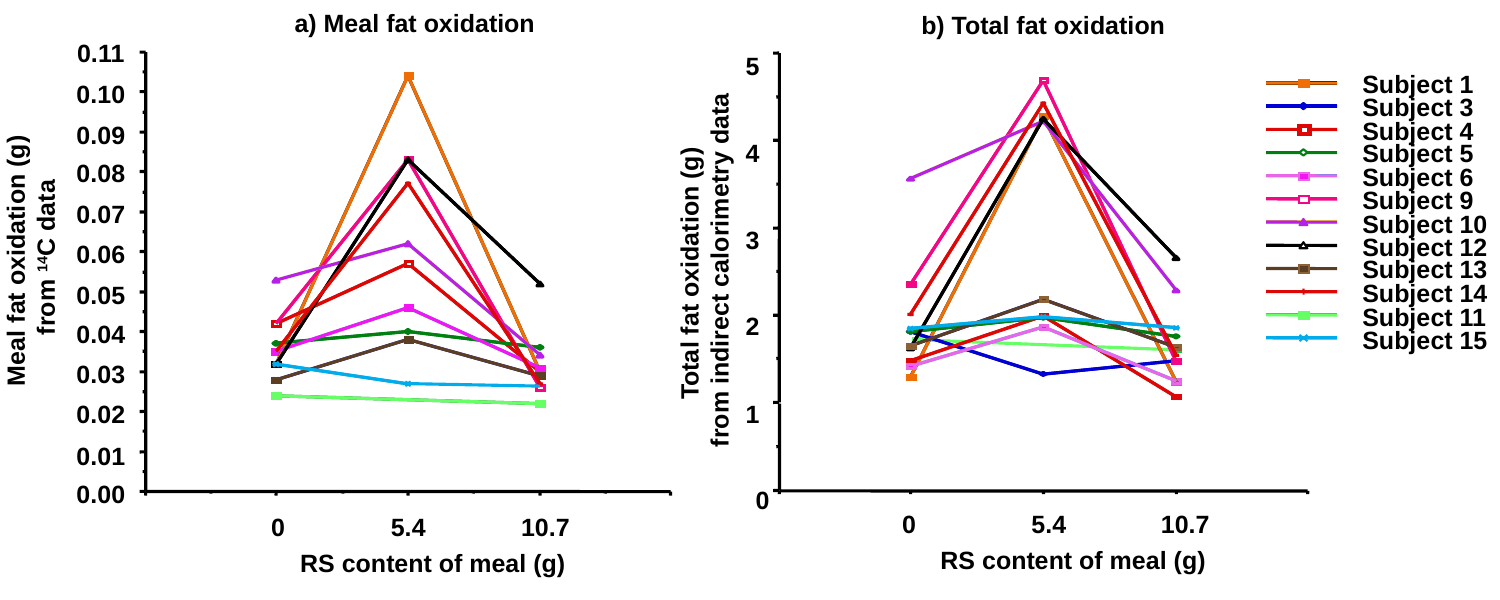

a) Meal fat oxidation
b) Total fat oxidation
0.11
0.10
0.09
0.08
0.07
0.06
0.05
0.04
0.03
0.02
0.01
0.00
Meal fat oxidation (g)
 from 14C data
0
5.4
10.7
RS content of meal (g)
5
4
3
2
1
Total fat oxidation (g)
 from indirect calorimetry data
0
0
5.4
10.7
RS content of meal (g)
Subject 1
Subject 3
Subject 4
Subject 5
Subject 6
Subject 9
Subject 10
Subject 12
Subject 13
Subject 14
Subject 11
Subject 15
