# Supplementary material for: Resistant starch consumption promotes lipid oxidation
Source: Nutr Metab (Lond). 2004 Oct 6;1:8. doi: 10.1186/1743-7075-1-8 (PMC526391; doi:10.1186/1743-7075-1-8)
Supplement: Additional File 2 — Individual area under the glucose curve vs. meal (a) and total fat oxidation (b) in response to a test breakfast. Meal fat oxidation, assessed via measurement of 14CO2 in expired air, and total fat oxidation, assessed via indirect calorimetry and calculated from non-protein RQ, and was measured in 12 healthy adults. Data from all three test meals (0%, 5.4%, and 10.7% RS) is shown. The relationship between area under the glucose curve and fat oxidation remains the same (i.e. no relationship) when represented as individual doses or, as in this plot, for all doses (see Figure S3). [file 1743-7075-1-8-S2.ppt]

## Slide 1
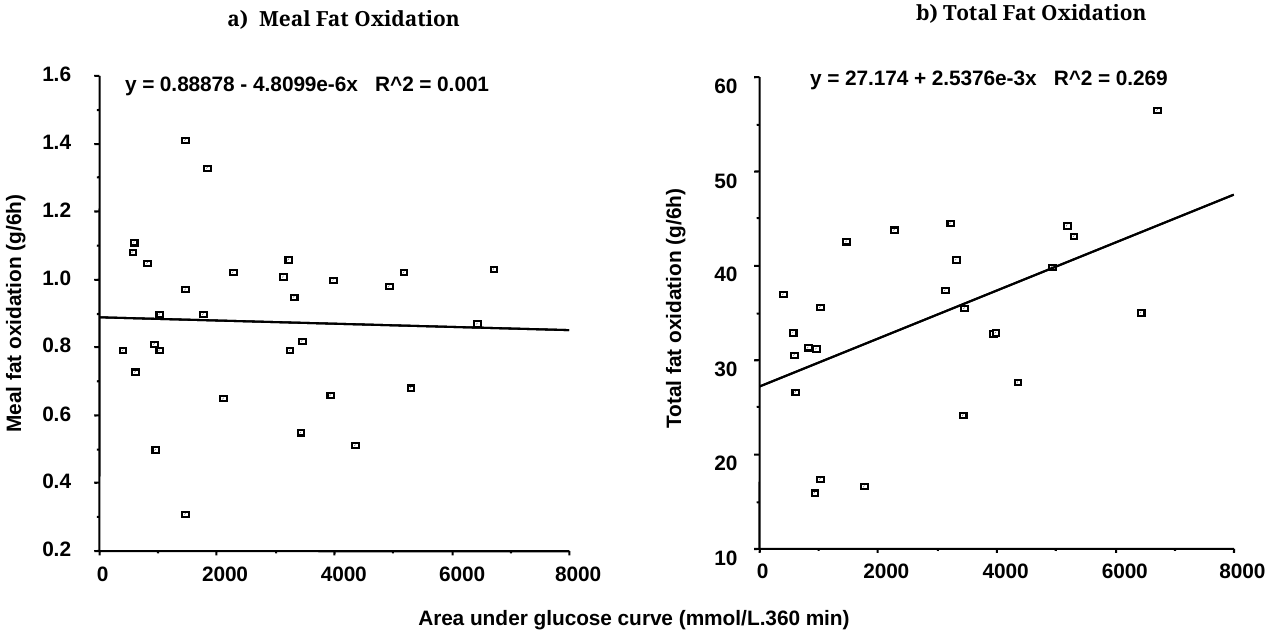

b) Total Fat Oxidation
a) Meal Fat Oxidation
1.6
1.4
1.2
1.0
0.8
0.6
0.4
0.2
y = 27.174 + 2.5376e-3x R^2 = 0.269
y = 0.88878 - 4.8099e-6x R^2 = 0.001
60
50
40
30
20
10
Total fat oxidation (g/6h)
Meal fat oxidation (g/6h)
0
2000
4000
6000
8000
0
2000
4000
6000
8000
Area under glucose curve (mmol/L.360 min)
