# Supplementary material for: Resistant starch consumption promotes lipid oxidation
Source: Nutr Metab (Lond). 2004 Oct 6;1:8. doi: 10.1186/1743-7075-1-8 (PMC526391; doi:10.1186/1743-7075-1-8)
Supplement: Additional File 3 — Individual area under the glucose curve vs. meal fat oxidation in response to a 0% (a) or 5.4% (b) RS test breakfast. Meal fat oxidation, assessed via measurement of 14CO2 in expired air, and total fat oxidation, assessed via indirect calorimetry and calculated from non-protein RQ, and was measured in healthy adults. Data from individual test meals is shown. [file 1743-7075-1-8-S3.ppt]

## Slide 1
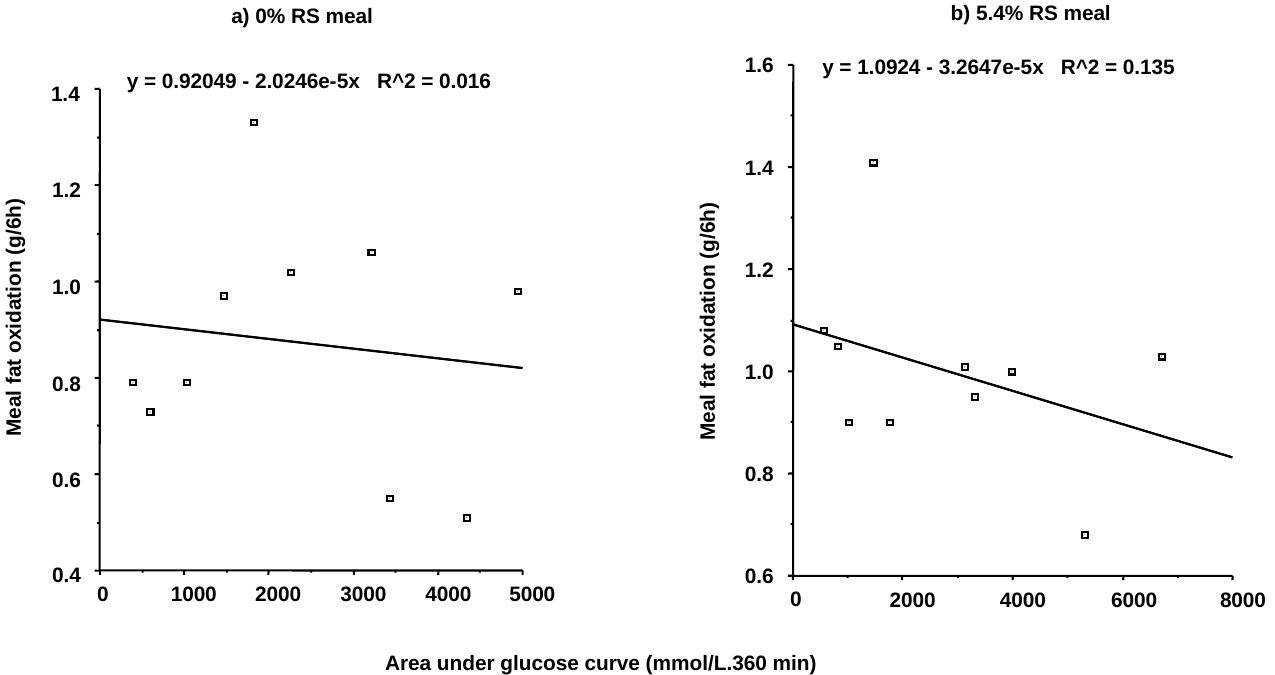

b) 5.4% RS meal
a) 0% RS meal
1.6
1.4
1.2
1.0
0.8
0.6
y = 1.0924 - 3.2647e-5x R^2 = 0.135
y = 0.92049 - 2.0246e-5x R^2 = 0.016
1.4
1.2
1.0
0.8
0.6
0.4
Meal fat oxidation (g/6h)
Meal fat oxidation (g/6h)
0
1000
2000
3000
4000
5000
0
2000
4000
6000
8000
Area under glucose curve (mmol/L.360 min)
