# Supplementary material for: Resistant starch consumption promotes lipid oxidation
Source: Nutr Metab (Lond). 2004 Oct 6;1:8. doi: 10.1186/1743-7075-1-8 (PMC526391; doi:10.1186/1743-7075-1-8)
Supplement: Additional File 4 — Individual area under the insulin curve vs. meal (a) and total fat oxidation (b) in response to a test breakfast. Meal fat oxidation, assessed via measurement of 14CO2 in expired air, and total fat oxidation, assessed via indirect calorimetry and calculated from non-protein RQ, and was measured in 12 healthy adults. Data from all three test meals (0%, 5.4%, and 10.7% RS) is shown. (Document type: Powerpoint, PPT) [file 1743-7075-1-8-S4.ppt]

## Slide 1
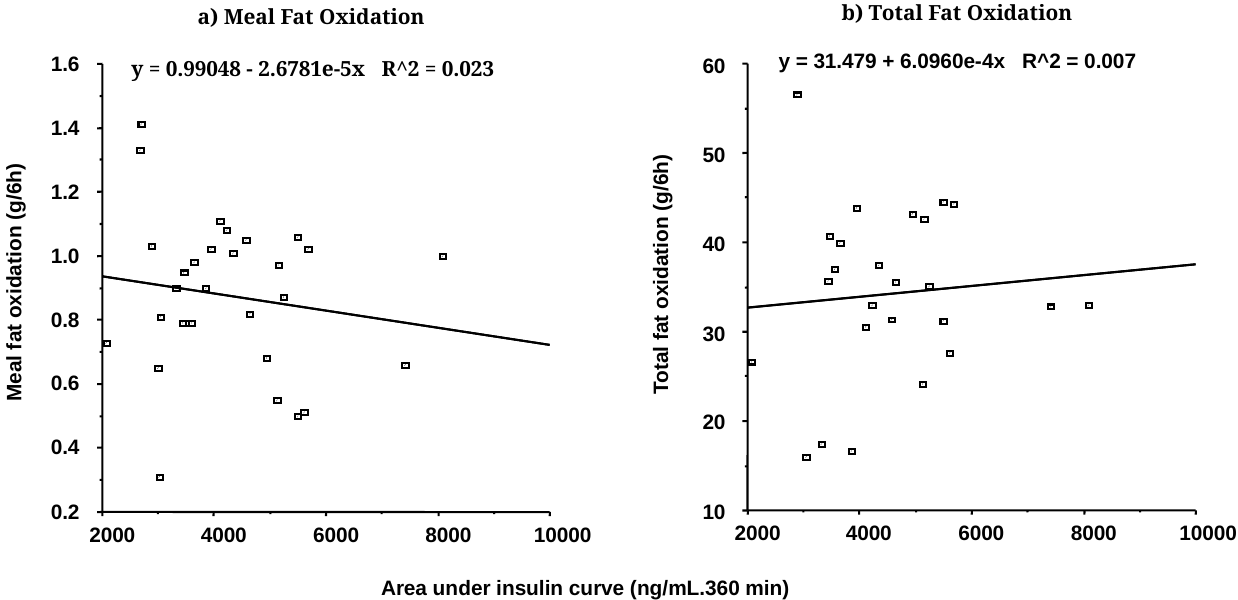

b) Total Fat Oxidation
a) Meal Fat Oxidation
y = 31.479 + 6.0960e-4x R^2 = 0.007
1.6
1.4
1.2
1.0
0.8
0.6
0.4
0.2
60
50
40
30
20
10
y = 0.99048 - 2.6781e-5x R^2 = 0.023
Total fat oxidation (g/6h)
Meal fat oxidation (g/6h)
2000
4000
6000
8000
10000
2000
4000
6000
8000
10000
Area under insulin curve (ng/mL.360 min)
